# Developing interventions to improve health: a systematic mapping review of international practice between 2015 and 2016

**DOI:** 10.1186/s40814-019-0512-8

**Published:** 2019-11-08

**Authors:** Liz Croot, Alicia O’Cathain, Katie Sworn, Lucy Yardley, Katrina Turner, Edward Duncan, Pat Hoddinott

**Affiliations:** 1 Medical Care Research Unit, School of Health and Related Research (ScHARR), University of Sheffield, Regent Court, 30 Regent Street, Sheffield, S1 4DA UK; 2 Population Health Sciences, University of Bristol, Bristol, UK; 3 NMAHP Research Unit, University of Stirling, Stirling, UK

**Keywords:** Complex intervention, Intervention development, Systematic mapping review

## Abstract

**Background:**

Researchers publish the processes they use to develop interventions to improve health. Reflecting on this endeavour may help future developers to improve their practice.

**Methods:**

Our aim was to collate, describe, and analyse the actions developers take when developing complex interventions to improve health. We carried out a systematic mapping review of empirical research studies that report the development of complex interventions to improve health. A search was undertaken of five databases over 2015–2016 using the term ‘intervention dev*’. Eighty-seven journal articles reporting the process of intervention development were identified. A purposive subset of 30 articles, using a range of published approaches to developing interventions, was selected for in-depth analysis using principles of realist synthesis to identify the actions of intervention development and rationales underpinning those actions.

**Results:**

The 87 articles were from the USA (39/87), the UK (32/87), continental Europe (6/87), and the rest of the world (10/87). These mainly took a pragmatic self-selected approach (*n* = 43); a theory- and evidence-based approach, e.g. Intervention Mapping, Behaviour Change Wheel (*n* = 22); or a partnership approach, e.g. community-based participatory research, co-design (*n* = 10). Ten actions of intervention development were identified from the subset of 30 articles, including identifying a need for an intervention, selecting the intervention development approach to follow, considering the needs of the target population, reviewing published evidence, involving stakeholders, drawing or generating theory, and designing and refining the intervention. Rationales for these actions were that they would produce more engaging, acceptable, feasible, and effective interventions.

**Conclusions:**

Developers take a variety of approaches to the international endeavour of complex intervention development. We have identified and described a set of actions taken within this endeavour regardless of whether developers follow a published approach or not. Future developers can use these actions and the rationales that underpin them to help them make decisions about the process of intervention development.

**Trial registration:**

PROSPERO, CRD42017080545.

## Strengths and limitations of this study

- This is a review of how intervention development is undertaken in a wide variety of contexts
- It is unlikely to include all intervention development studies published in 2015–2016 but does include the breadth of approaches to intervention development
- Principles of realist synthesis were used to identify rationales for actions taken during intervention development
- Journal articles are likely to be ‘cleaned-up’ versions of real-world practice

## Background

Researchers develop interventions to improve health. Research funding agencies invest in this phase of research (e.g. Medical Research Council Public Health Intervention Development Scheme https://mrc.ukri.org/funding/browse/public-health-intervention-development-scheme/public-health-intervention-development-scheme-phind-july-2017/), developers offer transparency by publishing the processes they have undertaken [1, 2], and scholars publish guides on how to develop interventions [3–6]. Hoddinott [7] welcomes the new era of intervention development studies, proposing the definition as ‘[a] study that describes the rationale, decision making processes, methods and findings which occur between the idea or inception of an intervention until it is ready for formal feasibility, pilot or efficacy testing prior to a full trial or evaluation’ (p. 1). Complex interventions, with multiple interacting components [8, 9], are often the focus of intervention development studies.

The UK’s Medical Research Council (UK MRC) produced internationally renowned guidance for the development and evaluation of complex interventions [8, 9]. It proposes four phases: development, feasibility and piloting, evaluation, and implementation. The first phase ‘development’ is where the ‘intervention must be developed to the point where it can reasonably be expected to have a worthwhile effect.’ (p. 2) [8]. This article focuses on this development phase but recognises it may not have a clear start and end point. Intervention development is of interest to readers of this journal because it so often overlaps with feasibility testing. It has been the subject of a special issue of Pilot and Feasibility Studies laying out the field- [7] and publishing-specific approaches [10, 11] and a subsequent overview of approaches [12]. As Hoddinott points out, there is often a grey area between this phase and the next phase of feasibility and piloting because some exploration of the early feasibility of delivering an intervention in a particular context is often part of the intervention development process [7].

There is also a grey area at the start of the intervention development phase. Prior to an intensive development phase, developers may undertake a series of activities over a number of years involving assessment of the evidence base, qualitative research or engagement with key stakeholders, or both. Alternatively, these activities may be undertaken as part of the intensive development phase.

A methodological review of published approaches to guide intervention development identified a taxonomy of 8 categories of approaches to intervention development [12] and 18 actions proposed within them. Practice can differ from the ideal scenarios recommended within guides so it is important to understand how developers undertake this endeavour in practice. This may highlight the aspects of intervention development that are not addressed by current guides or are promoted within guides but not used in practice. In addition, understanding the rationales for developing an intervention in a particular way may help to make transparent potential links between the way an intervention is developed and its subsequent success in terms of being acceptable, feasible, effective, implemented, and sustained in the real world [13]. Prior to undertaking this review, there was an evidence base on how intervention development is undertaken in practice. However, reviews were undertaken in specific contexts or for specific health conditions, e.g. optimisation prior to a randomised controlled trial [14], stroke care [15], and changing healthcare professional’s behaviour [16]. There was no evidence of how interventions that aim to improve health are developed across a range of contexts and conditions. The aim of this systematic mapping review was to collate and analyse the actions taken by developers, and describe the rationales for taking these actions, when developing complex interventions to improve health.

## Methods

### Design

We undertook this systematic mapping review as part of a wider study ‘IdentifyiNg and assessing different approaches to DEveloping compleX interventions’ (the INDEX study) [https://www.sheffield.ac.uk/scharr/sections/hsr/mcru/indexstudy]. A systematic mapping review is a way of collating studies to answer questions relating to the nature of evidence on a topic [17, 18]. Their purpose is to collate, describe, and catalogue evidence rather than to answer a specific question [17]. We undertook a systematic mapping review of empirical research studies reporting the development of complex interventions to improve health in order to understand the actions developers take in this endeavour. We were interested in the methods of the studies rather than their findings. Guidance on reviewing methods identifies that method-related sections of journal articles are a useful source of information about methods and how they are used [19]. We carried out a systematic search, screening, cataloguing, and analysis of the method-relevant sections of empirical research articles in two stages. The first stage focused on describing the characteristics of all the included articles and cataloguing them according to O’Cathain et al.’s eight categories of published approach taken, for example, partnership or theory- and evidence-based approaches [20]. The second stage involved selecting a purposive subset of articles ensuring coverage from different categories of published approaches [17].

The proposal was registered with the International Prospective Register of Systematic Reviews (PROSPERO) (registration number CRD42017080553, see Additional file 1). Preferred Reporting Items for Systematic reviews and Meta-Analyses (PRISMA) reporting guidelines were followed [21].

### Search strategy

We identified primary research studies using formal database searches. We searched MEDLINE, CINAHL, PsycINFO, ASSIA, and ERIC databases using the search term ‘intervention dev*’ with date parameters for the 2-year period from January 2015 to December 2016. We undertook this search in January 2017 and selected 2015–2016 to understand the recent practice. The full electronic search strategy for MEDLINE is presented in Additional file 2. This search produced 417 unique hits.

### Selection

The inclusion criteria were as follows:

- Journal articles reporting intervention development or planned development within published protocols
- Articles published between January 2015 and December 2016
- Interventions with a health-related outcome
- Development of a specific intervention, regardless of whether an intervention was produced or not
- Articles that reported a part of the intervention development process and positioned the work explicitly as the development of a specific intervention, for example, in their title
- Development activities occurring prior to a formal feasibility/pilot phase

The exclusion criteria were as follows:

- Publication types: methodological reviews (but the content of systematic reviews scanned for relevant studies)
- Articles describing simple rather than complex interventions, for example, development of drugs and devices, surgical procedures, biomedical screening, and those with non-human participants
- Articles detailing refinement of interventions during or following formal feasibility/pilot or evaluation phases
- Primary research which was not carried out with the explicit intention of developing an intervention

### Screening

KS screened all 417 titles and abstracts. AOC and LC double screened the first and last 25 abstracts taken from the retrieved records database. Any differences in opinion about inclusion were discussed and inclusion criteria refined. One hundred eighty-one articles were retrieved, and KS screened the full texts. Thirty-seven of these were double screened by AOC or LC. A total of 87 papers were included after discussion by AOC, LC, and KS, see Additional file 3 for the list of 87 articles.

### Quality assessment

In accordance with established methods for systematic mapping reviews, the quality of the studies was not assessed [17]. In addition, the focus of the analysis was the actions taken, not the findings of the studies.

### Cataloguing the studies

KS extracted information about the country of the first author, setting, and disease/condition into an electronic database for all 87 articles. KS, LC, and AOC categorised the approach used in each article using the taxonomy of 8 categories identified in a systematic methods overview of published approaches to intervention development [20]. A ninth category was identified from the 87 articles: ‘pragmatic’ where developers did not reference the use of any published approach but instead used a self-selected set of actions, sometimes framed as ‘formative evaluation’ or ‘mixed methods’. This 9-category taxonomy was used as a coding frame to guide our purposive sampling strategy to identify 30 articles for in-depth analysis of the actions of development and the rationales for those actions (see Table 1).

**Table 1:** Mapping the approaches used according to an intervention development taxonomy (see O’Cathain et al. [12]

	Category	Sub-category: specific approach described in an article	*N* = 87	Subset selected for analysis *n* = 30
1	Partnership	Community based participatory research	3	2
		Collaborative process	2	1
		Community engaged	2	1
		Others: co-produced, participatory ergonomics, concept mapping	3	3
2	Target population centred	User centric	1	0
3	Theory and evidence	MRC guidance	4	2
		Behaviour Change Wheel	4	1
		COM-B, Theoretical Domains Framework, or both	4	2
		Intervention Mapping	4	4
		Combinations within this category, e.g. Behaviour Change Wheel within MRC guidance	3	1
		Others, e.g. normalisation process theory, theory of change, behaviour change techniques	3	0
4	Implementation	–		
5	Efficiency	Experimental: multiple point baseline design	1	0
6	Stepped or phased	Five actions model	1	1
7	Intervention-specific	Digital	2	1
		For children/youth: deployment-focused model	1	0
8	Combination of categories	Examples include Behaviour Change Wheel and user-centred, or theory- and person-based approach	6	3
9	Pragmatic	No obvious framing	21	5
		Methods framing: mixed methods, qualitative, formative	10	2
		Partnership framing	4	1
		Theory and evidence framing	4	0
		Others, e.g. consensus	4	0

### Analysis of a subset of studies

A subset of 30 articles was selected purposively to maximise variation by selecting articles from different categories and sub-categories in an intervention development taxonomy (Table 1). The analysis of this subset was informed by the principles of realist reviewing [22]. Realist reviewing is a theory-driven approach that we selected to provide an explanatory analysis of intervention development processes. In a realist review, the unit of analysis is the explanation about how something works and the conditions that affect how it works [23]. Reviewers specify the context (C) and the outcome (O) of an intervention, along with the mechanisms (M) by which the intervention is assumed to affect the outcome. These relationships are often depicted in Context-Mechanism-Outcome Configurations (CMOCs) [24]. Typically, realist reviews focus on the mechanisms of interventions whereas our focus was on the processes of intervention development. For our review, the actions used to develop the intervention were equivalent to the context in a more traditional realist review (C), and the rationale for each action was the mechanism (M) by which developers hoped to improve the likelihood of success of the resultant intervention (O). For example, in one of our included articles, an intervention was developed in consultation with those targeted by the intervention (action/context) because the developers believed that interventions should be tailored to the specific needs of the particular population and setting to ensure a match between the needs of the target population and the intervention (rationale/mechanism) because this means that the intervention is more likely to be appropriate and acceptable, and therefore, the target population is more likely to engage with it, which means it is more likely to be successful (outcome) [25]. Authors did not always make their rationales explicit, and in these cases, we identified any implicit rationales for an action described in any parts of the articles. As the interventions were untested, it was not possible to determine the actual impact of the actions on the resultant success of the final intervention (outcome). Instead, we developed a set of statements that described the assumptions made by the developers.

In-depth data analysis began with four articles that reported using Intervention Mapping [3] as the approach to intervention development. We chose to start with Intervention Mapping because it provides a clear outline of six steps for intervention development [3]. The four articles reported all six steps between them, although not all articles described using all the steps. They also described how they took each action. We developed a preliminary thematic framework using these steps as actions of intervention development. LC extracted the actions, and how developers addressed these actions, into a Word table.

Next, we selected articles from the 10 categorised as using a partnership approach to intervention development because these offered a contrast to Intervention Mapping. Using an approach similar to constant comparison [26], LC compared the actions of development with those already in the framework, adding and refining the framework in discussion with AOC and KS. Early iterations of the framework included actions such as deciding on the health issue to be addressed and identifying antecedents of the problem. LC and KS then continued the data extraction independently for articles in the theory- and evidence-based category. Next, articles from the largest group labelled ‘pragmatic’ were selected and added to the framework. Finally, some articles from the smaller of the 9 categories were included. Dual extraction was undertaken on 13 articles in total, and the analysis was continually refined based on team discussions resulting in an inductively developed framework of the key actions taken by developers in practice during intervention development.

LC and KS documented CMOCs for each action in each article separately within a Word table. The CMOCs for each action were then synthesised and refined to create a smaller number of higher-level statements which summarised the rationales from these CMOCs for each action.

## Results

**Fig. 1:**
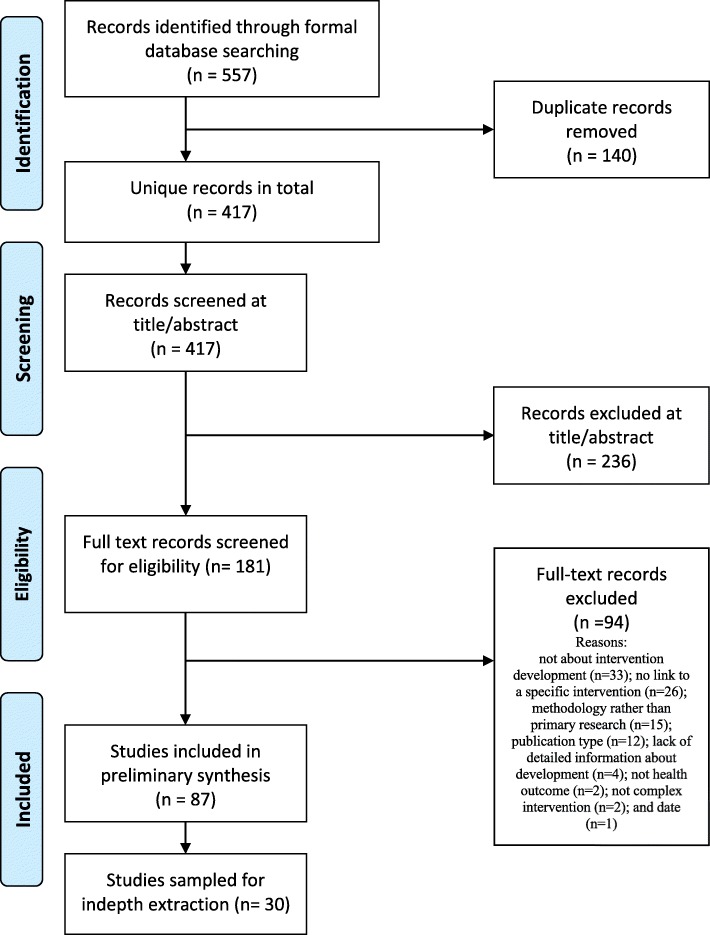
PRISMA diagram

Eighty-seven articles were included in the mapping review, and a subset of 30 articles was selected for further analysis (Fig. 1), see Additional file 3 for the list of included studies.

### Description of articles

The included 87 articles were written by lead authors based in the USA (39), the UK (32), other European countries (6), and the rest of the world (10). The majority (82%) were from the USA or the UK. The majority of the articles related to an intervention for a community setting including self-management (57). Other settings included hospital (19), general practice (8), school (5), workplace (3), prison (1), or military (1). Seven articles related to an intervention for more than one of these settings, for example, Webb et al. [27] developed an intervention targeting nurses working in either hospital or general practice settings. Many articles reported the development of digital interventions (30) including videos, web-based materials, and mobile phone applications. We classed the majority of these as community setting because the target population could choose to use them anywhere. Interventions focussed on a wide range of conditions including cancer (10), obesity/diet/weight management (11), mental health (10), sexual health (7), HIV (6), sedentary behaviour (6), substance misuse (5), and intimate partner violence (3). In some cases, interventions focussed on more than one condition, for example, Miller et al. [28] developed an intervention to improve the mental health of cancer survivors. A small number of interventions were aimed at health professional behaviour change to improve implementation and use of a new process, pathway, or intervention (5).

A wide range of approaches to intervention development was used, with a pragmatic approach accounting for half of the articles (Table 1). Allocation of articles to the taxonomy of approaches was straightforward for some sub-categories, such as Intervention Mapping, but challenging for articles that did not specify a referenced approach, that is, pragmatic sub-categories.

### Actions reported in a subset of 30 articles

We identified ten actions (see Table 2). Actions were reported in different orders in different articles.

**Table 2:** Summary of actions taken in intervention development and their rationales

Action	Rationales (based on the 30 articles in the subset)
1. Identify a need for an intervention	Interventions that meet a recognised health need are more likely to be implemented and used in the real world
2. Select the intervention development approach	Published approaches are systematic and transparent and therefore are more likely to lead to effective interventions
3. Consider the needs and circumstances of the target population	When interventions take into account the needs and circumstances of the target population, they are more likely to be accessible, acceptable, and relevant to that population, who then are more likely to engage and adhere to them
4. Determine the level(s) that the intervention will target, i.e. individual, organisational, environmental	Identifying the nature and potential impact of interactions between the intervention and different levels of influence allows the development of components to facilitate, mitigate, or mediate these interactions
5. Identify in-depth understanding of the setting for delivering the intervention	In-depth understanding of the setting is more likely to lead to credible solutions to delivering the intervention, which in turn may lead to an intervention that is feasible to implement in that setting, and therefore more likely to be implemented in the real world if found to be effective
6. Review published evidence on existing interventions	Research wastage associated with unnecessary new intervention development may be reduced if interventions are based on existing interventions or components shown to be effective in different contexts and adapted for a different health issue, population, or setting
7. Involve stakeholders	If relevant perspectives are used to shape the intervention, then it is more likely to be relevant, culturally appropriate, credible, and acceptable to those delivering or receiving the intervention, leading to implementation and engagement in the real world if found to be effective
8. Draw on existing theory and/or generate intervention-specific theory (programme theory)	Theory can help to identify relevant intervention components likely be effective in the target population, and it can illustrate how inputs produce outcomes to enable replication and evaluation of the impact of the intervention
9. Design the intervention	Designing an intervention that is feasible and acceptable to those delivering it and accessible to the target population leads to an intervention that is more likely to be implemented and used in the real world if found to be effective
10. Refine the intervention by assessing early feasibility and acceptability with stakeholders	Obtaining feedback on early versions of the intervention is more likely to produce a final intervention that is feasible and acceptable and therefore more likely to be used by the target population and implemented in the real world

#### Action 1: Identify a need for an intervention

Developers documented why a new intervention was needed through reference to the research literature [29], healthcare policy [10], their own expertise in research [30] or practice [31], previous research findings [32], consultation with community members [33], or a combination of these [34]. The rationale for this was to justify the development of a new intervention by identifying an unmet health need. In most cases, developers selected the health issue to focus on, but some developers used partnership approaches with communities to identify and prioritise health issues. For example, Njeru et al. [35] were part of a long-established community-academic partnership. This partnership had developed a community-based research infrastructure to enable community and academic partners to conduct every phase of research together. They identified type II diabetes as a priority area for intervention.

Identifying the health issue was important to establish the type of intervention needed. In some cases, no effective interventions existed for that health issue, and a new intervention was identified as necessary. For example, Theeke and Mallow [36] recognised the link between loneliness and poor health but noted existing loneliness interventions showed limited effectiveness and sustainability, so a new intervention was needed. In other cases, an effective intervention existed but was not implemented by practitioners, so an intervention to address implementation was needed. For example, Connell et al. [37] identified intensive repetitive task-orientated training as an effective intervention to promote recovery of upper limb function following a stroke but noted it was not carried out in practice, so they set out to develop an intervention to improve its implementation.

#### Action 2: Select the intervention development approach

Developers who used published approaches to intervention development often justified this in the introduction of their articles. Their explicit rationales for using published approaches were that they provided systematic and transparent processes for combining theory, published evidence, and new data. For example, Heath et al. [38] selected Intervention Mapping (Bartholomew et al. [39]) because it is ‘a problem-driven approach which combines one or multiple theories, empirical evidence and new research to develop behaviour change interventions’ (p. 1229).

When developers took a pragmatic approach, that is, did not follow a published approach, they framed it in diverse ways. Sometimes, the descriptions of the approach were inconsistent throughout the article, showing the challenge of describing approaches clearly. For example, Marsac et al. [40] described incorporating evidence-, theory-, and a user-centred design and referred to this as ‘systematic, theoretically grounded development’ in their title (p. 12) and ‘systematic and empirically grounded (p. 16) in the body of their article. Other developers sometimes drew on wider research designs rather than intervention development approaches to offer a set of steps for their approach to intervention development because this offered a systematic and transparent description of their approach. For example, Golin et al. [41] cited Linnan and Steckler’s [42] work on process evaluation as a source of best practice for intervention development and then presented a diagram of three phases and steps they took.

#### Action 3: Consider the needs and circumstances of the target population

Most developers identified the target population in the introduction section of their articles. The rationale for identifying the target population at an early stage was to allow developers to consider the needs and circumstances of that population in order to ensure the subsequent intervention was accessible, acceptable, and relevant to them. Developers identified the target population by referring to research literature highlighting a problem for that specific population and by referring to their previous research (e.g. Martin et al. [32]), national or international policy (e.g. Morrison et al. [43]), or pragmatic reasons for their decision to target a particular population. For example, Golin et al. [41] described the pragmatic reasons related to access to the population and potential reach of the intervention, which led them to target their intervention to a demographically diverse group of HIV-infected, English-speaking men and women, in 2 months before and 3 months after release from prison. In contrast, a small number of articles identified the target population during the development process. For example, Cadogan et al. [44] developed three draft interventions to improve appropriate polypharmacy in older people in primary care targeting patients, general practitioners, and community pharmacists. The research team then screened each draft intervention using the Affordability, Practicability, Effectiveness and cost-effectiveness, Acceptability, Side effects/safety and Equity (APEASE) criteria [45] and selected general practitioners as the target population for the planned intervention. When the intervention aimed to increase implementation of an existing intervention, the target population was not those directly affected by the health issue. For example, Steinmo et al. [46] set out to improve the implementation of the ‘Sepsis Six’ care bundle [47] because the target for use of the Sepsis Six bundle had not been reached within a UK hospital. In this instance, they viewed the target population as those in a position to implement ‘Sepsis Six’ rather than patients receiving the bundle.

#### Action 4: Determine the level(s) that the intervention will target, i.e. individual, organisational, and environmental

Some developers determined the level(s) the intervention would influence at the outset, stating this in the introduction of the article, whereas others did this during the development process. The rationale for selecting the intervention level(s) was to guide the choice of components and to consider the interplay between these. For example, Mackenzie et al. [25] cited Gilson et al.’s [48] suggestion that interventions should address multiple levels of influence, and set out to develop a multi-level intervention to reduce workplace sitting in a university department. They carried out a focus group with 13 members of their target population to identify appropriate intervention content to address individual, social, environmental, and organisational levels of influence. Some developers identified multiple levels of influence but chose to focus on only one of these levels. Ingholt et al. [49] developed an intervention to improve students’ social relations in order to reduce dropout from vocational schools along with smoking and drug use. They identified existing interventions targeting individual students but found that little attention had been given to schools themselves, and so, they chose to focus their intervention at the organisational level of school.

#### Action 5: Identify in-depth understanding of the setting for delivering the intervention

The rationale for identifying the setting for delivery was to allow developers to explore opportunities and constraints that could affect the feasibility and acceptability of delivering or receiving an intervention in that setting. The decision to develop an intervention for a particular setting was usually described in the introduction section of articles. This decision was based on the following: the location of the target population, for example, an intervention targeting recently incarcerated individuals with HIV was set in prisons and community-based HIV services [41]; the preferences of the target population, for example, Poleshuck et al. [50] used a participatory approach in which the members of the target population identified an existing women’s health clinic as the setting for their intervention to improve the health of survivors of intimate partner violence; a gap in the evidence base, for example, a lack of weight management programmes delivered solely by GPs in primary care in Australia [10]; or the interests and expertise of the research team, for example, Ingholt et al. [49] had previously completed a fieldwork in vocational schools in Denmark.

In some cases, the setting was a critical factor in identifying potential interventions. For example, Rothman and Wang [31] set out to develop an intervention to reduce dating abuse perpetration to be delivered in a hospital setting. They identified a brief, motivational interview-style intervention shown to be effective in reducing youth alcohol and marijuana use in a hospital setting and decided to adapt this to reduce adolescent dating abuse in the same setting. In some articles, the setting for delivery was not relevant, for example, when developers were producing an online intervention [51].

#### Action 6: Review published evidence on existing interventions

The rationale for this was to reduce research wastage associated with unnecessary intervention development. Many developers reviewed the existing literature in the introduction section of articles to establish the need for a new intervention [40] or to identify effective interventions, or components of existing interventions, that might be adapted or used for a different health issue [52], target population [29], or setting [53]. In a small number of articles, developers began by exploring the health issue in more detail to identify contributory or causal processes or pathways before searching the literature for existing interventions, or components of interventions, to address these. For example, Charles et al. [54] described the early-stage development of an intervention to improve psychosocial and behavioural health outcomes amongst children with fathers with a history of incarceration. They began by reviewing the literature to understand the health issue in more detail and made three observations. First, that the complex characteristics and needs of fathers should act to increase, not preclude, calls for a supportive service. Second, that developing fathers’ knowledge and parenting skills would be central to any intervention. Third, that fathers’ involvement could be improved by explicitly involving mothers and extended family in intervention efforts. They then reviewed the literature to select programme components from two manualised, previously evaluated interventions that addressed these observations.

#### Action 7: Involve stakeholders

The rationale for involving stakeholders was to enable developers to consider different perspectives in order to improve the potential fit between the intervention and the contexts in which it would be used. Stakeholders were people with interest, influence, expertise, or other concern in the intervention, health problem, target population, or setting. The examples included health and social care providers [55], academics [54], representatives of third sector organisations [32], members of the target population [25], policymakers [50], and advocates and gatekeepers to the target population [56]. Decisions reported about stakeholder involvement included who to include, the duration of involvement, and the nature and purpose of the involvement.

Developers reported working with stakeholders in different ways. Some established stakeholder groups from the outset or included stakeholders in the development team, so that they were involved in decisions about the overall intervention development endeavour for the duration of the process. Others consulted one or more groups of stakeholders episodically during the development of the intervention. Poleshuck et al. [50] did both. They convened an executive committee of researchers, representatives of the target population, and service providers to guide and oversee the intervention development endeavour throughout the process. This group then convened a community advisory board made up of survivors of violence (members of the target population), health professionals, academics, and policymakers to contribute to the design and content of their intervention. In this example, the executive group oversaw how the intervention would be developed, and the community advisory board brought a range of perspectives to discussions about the content, format, and delivery of the intervention at particular stages.

Some stakeholder groups included members of the target population—those at whom the intervention was aimed—and others did not. For example, Vaughn et al. [34] collaborated with the members of the target population in their ‘community-engaged’ study to identify strategies to address obesity, stress, and coping amongst the Latino immigrant community in Ohio. They stated that this engagement with the target population was necessary to develop strategies that were contextually and culturally appropriate. Developers working with high levels of stakeholder involvement identified a concern that the developed intervention might have limited transferability beyond the setting or group that contributed to its development. For example, Steinmo et al. [46] observed that their collaborative approach was an important strength and a significant lever to the success of the intervention because it allowed the intervention to be shaped to the specific context; however, it was also a limitation because they could not draw conclusions about feasibility in other hospitals.

#### Action 8: Draw on existing theory and/or generate intervention-specific theory

Developers referred to theory in different ways. Some identified existing mid-range theories (see Davidoff et al. [23] for definition) in the introduction of the article, for example, social practice theory [49], social-ecological theory [34], and the theory of planned behaviour [31]. The rationale for drawing on existing theory was that this would lead to interventions that were more likely to be effective. Developers of behaviour change interventions sometimes referred to frameworks of theories, e.g. the Behaviour Change Wheel [45] because they provided a systematic way of analysing behaviour and a comprehensive list of behaviour change techniques to consider [29, 44]. The rationale for this was to bring transparency to the process of development and allow for accurate replication and evaluation of the intervention mechanisms [53, 55].

Theory generation was an alternative or additional approach to using existing theory. Some developers described how they synthesised evidence from different sources to develop a programme theory specific to their intervention (e.g. Ford et al. [57]). The rationale for the intervention-specific theory was that theories should be tailored to the context in which they are being used. Developers used different methods to gather evidence to inform this programme theory. Many carried out systematic reviews of the literature to explore the health problem (e.g. Gray-Burrows et al. [58]). Others identified behavioural targets through consultation with stakeholders (e.g. Steinmo et al. [46]), from qualitative research with the target population (e.g. Mummah et al. [59]), or from a combination of all of these (e.g. Simonsen et al. [33]). Programme theory generated during development was sometimes articulated in a logic model specific to the intervention (e.g. Golin et al. [41]).

The weight given to a theory differed between articles. At the outset, some developers gave a detailed account of how the chosen theories or frameworks would inform development, whereas others gave little attention to this. For example, Golin et al. [41] described how the core tenets of social cognitive theory [60] and the information-motivation-behavioural skills model (Fisher et al. [61]) related to antiretroviral adherence in recently incarcerated individuals. They supported their choice of theory by referring to existing empirical evidence of the effectiveness of similar interventions, and later, they used a conceptual model to show the integration of these theories into their intervention. In contrast, Katz and Paskett [56] described their intervention as ‘theoretically based’ (p. 445) but referred to theory in just one sentence. Some developers did not refer explicitly to the theory at all, articulating some of their programme theory without using the term theory.

The point at which developers selected an existing theory varied. In some cases, developers selected an existing theory or theories prior to starting the development process whereas others began development and identified relevant theory as part of this process. For example, Vaughn et al. [34] describe how their study was ‘based on a framework of social-ecological theory’ (p. 838) [62], and this informed their work to integrate multiple perspectives to understand the contextual and cultural nuances of their target population. In contrast, Theeke and Mallow [36] set out to develop an intervention to target the cognitive processes associated with loneliness. They carried out a literature review about loneliness and identified two distinct intervention targets: the dysfunctional thinking associated with loneliness and the lack of a meaningful role described by people with loneliness. The team then selected theories that could be used to develop and implement an intervention to target these cognitive processes.

#### Action 9: Design the intervention

Developers made decisions about the components of the intervention and how to present and deliver these components, thinking about who would deliver them, how often, and for how long. The rationale for this was that interventions were more likely to be successful if the target population can access and engage with them, and they are more likely to be implemented if they are feasible and acceptable to those who will deliver them. Some developers made some of these decisions at the outset. For example, Smith et al. [63] chose to develop a mobile cancer prevention application targeting African-American breast cancer survivors because African-Americans have the highest smartphone ownership of all ethnic groups. In contrast, other developers identified the mode of delivery during intervention development. For example, Gray-Burrows et al. [58] developed a logic model for their intervention to reduce dental caries by promoting parental-supervised tooth brushing and then identified programme components and modes by which these could be delivered within the existing provision. Some developers took a creative approach to identifying options for the intervention, engaging in group discussion sessions they called ‘ideation’ [59].

Some interventions required technical expertise to produce the intervention. In some cases, the developers generated the intervention content and used third party technical expertise to design the resultant intervention product. For example, Sturgiss and Douglas [10] employed a graphic designer to improve the usability and attractiveness of the documents they produced for general practitioners using their intervention. In contrast, Mummah et al. [59] included technical developers within their development team to integrate design thinking throughout the development process.

#### Action 10: Refine the intervention by assessing early feasibility and acceptability with stakeholders

Many interventions were refined during development, iteratively and collaboratively with target users and other stakeholders before the formal feasibility and piloting stages. The rationale was that incorporating the views and preferences of stakeholders on early versions would improve the feasibility and acceptability of the resultant intervention. Developers producing digital interventions tested prototypes with users during development specifically to assess functionality (readability, ease of navigation), engagement, and acceptability. For example, Marsac et al. [40] produced a prototype of their digital intervention ‘Coping Coach’ for children affected by acute medical trauma, which they tested with users, refined based on this test, and then retested. A number of technical difficulties were identified with prototype 1, and major revisions were made to produce prototype 2. Small numbers of the target population assessed the first prototype (*n* = 9), and larger numbers tested the second prototype (*n* = 33). The authors stated that by systematically evaluating the intervention during the development process, they could optimise the design based on user feedback and reduce time and development costs. Developers assessed early feasibility and acceptability by working with a stakeholder group or by carrying out qualitative or quantitative research with members of the target population, those who would be delivering the intervention, or both. Some developers tested prototypes or drafts of individual components of the intervention (Marsac et al. [40]) whereas others tested the whole intervention, resulting in refinements to both the intervention and its delivery (e.g. McMillen et al. [64], Sturgiss and Douglas [10]), before moving on to feasibility and pilot testing.

### Rationales

For each action, rationales within the 30 articles were identified and summarised within a single statement (see Table 2).

## Discussion

### Summary of findings

Publishing how interventions were developed is a common and international endeavour. Developers take very different approaches, including following published approaches or determining their own set of actions. Ten actions of intervention development were identified from the subset of 30 papers, including identifying a need for an intervention, selecting the intervention development approach to follow, considering the needs of the target population, reviewing published evidence, involving stakeholders, drawing on theory, designing the intervention, and refining the intervention. Few articles reported all the actions undertaken, and a small number covered only 1 or 2 actions in considerable detail. Developers also gave different weight to the actions they reported. Rationales for these actions were that they would produce more acceptable, feasible, and effective interventions.

### Strengths and limitations

The strength of this work lies in the detailed account of the reported actions taken during intervention development and the rationales for those actions, drawing on real-world practice. There were 6 limitations. First, the search term used to identify studies was simple and may not have identified all reports of intervention development published in this timeframe. To consider the effect of this, we undertook a second search with a wider range of terms in the same databases: complex behavioural intervention, develop, design, phase 1, exploratory, refine, and translate. We selected the first 100 records and then 1 in every 8 records of the 808 records retrieved. We conducted a title and abstract screen on these 189 records and identified 26 relevant articles. This did not identify further categories or sub-categories of intervention development reported in Table 2. Second, we did not search the grey literature, and doing so may have identified accounts of interventions not published in an academic journal. Third, this is a rapidly developing field, and although the included articles were published in 2015 and 2016, the development activity took place earlier than this, and so published accounts may not fully represent recent innovation in this area. Fourth, it was important to sample from each category which we did; however, it is possible that by not sampling from each sub-category, we may have missed an action. In addition, a different research team might have purposively selected a different subset of 30 papers for in-depth analysis. For example, we noticed in hindsight that we did not include 2/87 articles from lower- and middle-income countries in the in-depth analysis. Fifth, we attempted to identify the rationale for undertaking actions. It is important to note that these were assumptions and we did not follow up the interventions to assess whether the interventions developed had indeed been effective. A recent systematic review attempted to examine the link between the use of one of the actions in our analysis—theory—and the effectiveness of the subsequent interventions and concluded that the use of theory was not associated with effective interventions [65]. Therefore, we offer these actions and rationales as hypotheses for future testing. Finally, although we present the findings to illustrate real-world practice, researchers often have to offer cleaned-up versions of their practice and may not always document everything in their papers so that readers can easily understand the content and reporting fits the template for articles within specific journals.

### Placing the research in the context of other evidence

There was considerable overlap between the 10 actions identified here and 18 actions described in a systematic methods overview of published approaches to intervention development [20]. There were differences in how the actions in the different reviews were described, rather than actual differences between the sets of actions. Some actions appeared in this review that would not be expected to appear in actions based on published approaches, in particular, selecting which published approach to take. Our findings were similar to those of other researchers who had reviewed practice in the more specific areas of nursing [66], changing health professionals’ behaviour [13], and behaviour change for chronic conditions [67].

## Conclusion and implications

Within this mapping review, we described actions taken in practice by those developing interventions in a range of contexts, illustrating them with examples. The review adds to a growing body of evidence identifying a similar set of actions in specific contexts. Many developers did not follow guidance encapsulated in published approaches to intervention development but nonetheless undertook actions recommended by those approaches. Developers varied in how they undertook these actions. This mapping review of practice will be useful to those planning intervention development so they can understand how others have done this and the rationales for the choices they have made. We plan to use the findings from the review together with other evidence to construct guidance on how to develop interventions. There remains a knowledge gap about the relationship between these actions and the success or otherwise of the resultant interventions.

## Supplementary information

**Additional file 1.** Prospero protocol.

**Additional file 2.** Search strategy.

**Additional file 3.** Full list of 87 included studies.

## Data Availability

The datasets used during the current study are available as additional files.
