# Supplementary material for: Developing interventions to improve health: a systematic mapping review of international practice between 2015 and 2016
Source: Pilot Feasibility Stud. 2019 Nov 8;5:127. doi: 10.1186/s40814-019-0512-8 (PMC6839208; doi:10.1186/s40814-019-0512-8)
Supplement: Supplementary file 1 — Additional file 1. Prospero protocol. [file 40814_2019_512_MOESM1_ESM.pdf]

A methodological systematic review of the development of complex interventions aimed at improving health outcomes

Alicia O′ Cathain, Elizabeth Croot, Katie Sworn

Citation

Alicia O′ Cathain, Elizabeth Croot, Katie Sworn. A methodological systematic review of the development of complex interventions aimed at improving health outcomes. PROSPERO 2017 CRD42017080553 Available from:
http://www.crd.york.ac.uk/PROSPERO/display_record.php?ID=CRD42017080553

Review question

What approaches to developing interventions are there?
What are the rationales for taking different approaches?
What are the core principles and practices of intervention development?
What contexts are approaches used within?
What research methods are used at different steps of each approach?
What are the strengths and weaknesses of each approach?
Note: Our review approach is broad as we are looking at all health-related complex interventions in a way that cuts across all health conditions and outcomes.

Searches

Sources will be identified from formal and informal database search techniques. Our review focus will be sources published in the last 10 years. We will perform up to three iterations of database searches in MEDLINE, CINAHL, PsycINFO, ASSIA and ERIC databases. The formal database searches will identify the most recent papers published January 2015- December 2016.
Iteration 1 will use the term ′intervention development′. Iteration 2 will include wider range of terms about intervention development (′complex behavioural intervention′) and the development stages (develop, design, phase I, exploratory, refine and translate). Subsequent iterations will be contingent on results of the previous iterations. If the subsequent iterations appear to have low specificity a sample may be screened for relevant papers.
Methodological sources acquired for this review will be retrieved through informal forms of searching such as Google Scholar, the reference libraries of team members and reference searching of included studies. Terms used included ′intervention development′, ′complex intervention development′, ′intervention optimisation′, ′complex intervention pre-clinical′, ′intervention adaptation′ and ′intervention modification′. Sources will include: core methodological books, guidance and methodologies provided in papers within key journals.
Searching will employ a maximum variation approach in order to present the widest range of methodologies possible. However, searches will be comprehensive, not exhaustive. Searching will cease when the research team judge they have reached theoretical saturation.

Types of study to be included

Inclusion and exclusion criteria for the searches:
Inclusion criteria

- International complex intervention methodological sources.
- Published material from a number of sources (books, papers, guidelines or frameworks).
- Methodological material must have potential to be applied to the development of complex intervention with health-related outcomes

- Focus on planning and design phases prior to full feasibility and acceptability
- testing. Methodologies do not have to be proven to increase successfulness of

  interventions.

Exclusion criteria

- Methodologies applied exclusively to simple interventions involving efficacy of drugs or
- procedures. Refinement of interventions at the pilot or full randomised controlled trial phase and
- beyond. Materials not translated into English.

Condition or domain being studied

The domain being studied is approaches to developing complex interventions.

Participants/population

This review encompasses methodological materials to be used in complex intervention development for the recipients of any intervention which has a health-related outcome.

Intervention(s), exposure(s)

The complex interventions might work at any level: individual, group or multi-level.

Comparator(s)/control

Not applicable

Context

The contexts of the development methodologies are likely to be health or health-related disciplines. The context of the studies in which the methodologies are applied could be extremely diverse across international health settings. Methodologies from educational settings will be considered where health-related outcomes could be possible in development studies.

Main outcome(s)

The outcome of the review is to identify and analyse methodological approaches and associated methods to inform guidance for complex interventions developers.

Timing and effect measures

Not applicable

Additional outcome(s)

Not applicable

Timing and effect measures

Not applicable

Data extraction (selection and coding)

Methodological sources will be gathered into an electronic library and reference management software. Data extraction will take place using tables produced in Word. The reviewers will differentiate between data derived from the sources and interpretations made by the review team. The reviewer will link methodologies

to broad methodological categories. Other data extraction variables will relate to methodological and methods characteristics, such as: approaches, author, rationale, context and steps. The reviewer will extract any strengths and limitations provided by the methodologists.

Risk of bias (quality) assessment

No formal assessment of methodological quality will take place and no methodological source will be excluded on the basis of quality (e.g. evidence of take up in the literature base). The appraisal of the strengths and weaknesses of the methodologies is part of the analytical remit of the review.

Strategy for data synthesis

The constant comparison method will be applied to the extracted data (Gentles, 2016). Synthesis will generate a list of principles and practices of intervention development. The reviewers will create their own interpretation of the strengths and limitations of methodologies and these interpretations will be discussed with the review team and wider study team.

Analysis of subgroups or subsets

None planned

Contact details for further information

Katie Sworn
k.sworn@sheffield.ac.uk

Organisational affiliation of the review

University of Sheffield
https://www.sheffield.ac.uk

Review team members and their organisational affiliations

Professor Alicia O′ Cathain. University of Sheffield)
Dr Elizabeth Croot. University of Sheffield
Dr Katie Sworn. University of Sheffield

Collaborators

Dr Edward Duncan. University of Stirling
Professor Pat Hoddinott. University of Stirling
Dr Katrina Turner. University of Bristol
Professor Lucy Yardley. University of Southampton
Dr Nikki Rousseau. University of Stirling

Anticipated or actual start date

01 April 2016

Anticipated completion date

31 March 2018

Funding sources/sponsors

UK Medical Research Council

Conflicts of interest

Language

English

Country

England

Stage of review

Review_Ongoing

Subject index terms status

Subject indexing assigned by CRD

Subject index terms

Health Promotion; Humans; Program Evaluation/methods; Public Health

Date of registration in PROSPERO

28 November 2017

Date of publication of this version

28 November 2017

Details of any existing review of the same topic by the same authors

Stage of review at time of this submission

Stage	Started	Completed
Preliminary searches	Yes	Yes
Piloting of the study selection process	Yes	Yes
Formal screening of search results against eligibility criteria	Yes	Yes
Data extraction	Yes	No
Risk of bias (quality) assessment	Yes	No
Data analysis	Yes	No

Versions

28 November 2017

PROSPERO
This information has been provided by the named contact for this review. CRD has accepted this information in good faith and registered the review in PROSPERO. CRD bears no responsibility or liability for the content of this registration record, any associated files or external websites.
