# Supplementary material for: Developing interventions to improve health: a systematic mapping review of international practice between 2015 and 2016
Source: Pilot Feasibility Stud. 2019 Nov 8;5:127. doi: 10.1186/s40814-019-0512-8 (PMC6839208; doi:10.1186/s40814-019-0512-8)
Supplement: Supplementary file 2 — Additional file 2. Search strategy. [file 40814_2019_512_MOESM2_ESM.pdf]

Supplementary file 2 Search strategy

Primary search

MEDLINE Epub Ahead of Print, In-Process & Other Non-Indexed Citations, Ovid MEDLINE(R) Daily and Ovid MEDLINE(R)
3rd January 2017

#	Searches
1	intervention development.ti,ab.
2	limit 1 to english language
3	limit 2 to yr="2015 - 2016"

CINAHL
3rd January 2017

#	Searches
1	TI intervention development OR AB intervention development Limiters - Published Date: 20150101-20161231; English Language

PsycINFO
3rd January 2017

#	Searches
1	intervention development.ti,ab.
2	limit 1 to english language
3	limit 2 to yr="2015 - 2016"

ASSIA
3rd January 2017

#	Searches
S1	ti("intervention development") OR ab("intervention development") Additional limits - Date: 2015-2016; Language: English

ERIC
3rd January 2017

#	Searches
S1	ti("intervention development") OR ab("intervention development") Additional limits - Date: 2015-2016; Language: English

Wider search to check breadth of primary search

MEDLINE Epub Ahead of Print, In-Process & Other Non-Indexed Citations, Ovid MEDLINE(R) Daily and Ovid MEDLINE(R)
3rd January 2017

#	Searches
1	((complex or behav?oural) adj3 intervention*).ti,ab.
2	develop*.ti,ab.
3	design*.ti,ab.
4	clinical trial, phase i.pt.
5	(phase adj ('1' or I or first or one)).ti,ab.
6	refine*.ti,ab.
7	translat*.ti,ab.
8	exploratory.ti,ab.
9	or/2-8
10	1 and 9
11	limit 10 to yr="2015 - 2016"
12	limit 11 to english language

CINAHL
3rd January 2017

#	Searches
S1	TI (((complex or behav?oural) N3 intervention*).) OR AB (((complex or behav?oural) N3 intervention*))
S2	TI ((develop* or design* or refine* or translat* or exploratory)) OR AB ((develop* or design* or refine* or translat* or exploratory))
S3	TI ((phase N1 ('1' or I or first or one))) OR AB ((phase N1 ('1' or I or first or one)))
S4	S2 OR S3
S5	S1 AND S4
S6	S1 AND S4 Limiters - Published Date: 20150101-20161231; English Language

PsycINFO
3rd January 2017

#	Searches
1	((complex or behav?oural) adj3 intervention*).ti,ab.
2	develop*.ti,ab.
3	design*.ti,ab.
4	(phase adj ('1' or I or first or one)).ti,ab.
5	refine*.ti,ab.
6	translat*.ti,ab.
7	exploratory.ti,ab.
8	or/2-7

9	1 and 8
10	limit 9 to yr="2015 -2016"
11	limit 10 to english language

ASSIA
3rd January 2017

#	**Searches**
S1	(ti(((complex OR behav?oural) NEAR/3 intervention*)) OR ab(((complex OR behav?oural) NEAR/3 intervention*))) AND ((ti(develop* OR design* OR refine* OR translat* OR exploratory) OR ab(develop* OR design* OR refine* OR translat* OR exploratory)) OR (ti((phase N ('1' OR I OR first OR one))) OR ab((phase N ('1' OR I OR first OR one))))) AND (la.exact("ENG") AND pd(20150101-20161231))

ERIC
3rd January 2017

#	**Searches**
S1	(ti(((complex OR behav?oural) NEAR/3 intervention*)) OR ab(((complex OR behav?oural) NEAR/3 intervention*))) AND ((ti(develop* OR design* OR refine* OR translat* OR exploratory) OR ab(develop* OR design* OR refine* OR translat* OR exploratory)) OR (ti((phase N ('1' OR I OR first OR one))) OR ab((phase N ('1' OR I OR first OR one))))) AND (la.exact("ENG") AND pd(20150101-20161231))
