# Supplementary material for: Developing interventions to improve health: a systematic mapping review of international practice between 2015 and 2016
Source: Pilot Feasibility Stud. 2019 Nov 8;5:127. doi: 10.1186/s40814-019-0512-8 (PMC6839208; doi:10.1186/s40814-019-0512-8)
Supplement: Supplementary file 3 — Additional file 3. Full list of 87 included studies. [file 40814_2019_512_MOESM3_ESM.pdf]

Supplementary file 3, 87 included papers (* paper included in subsample)

1. Arnold EA, Operario D, Cornwell S, Benjamin M, Smith CD, Lockett G, Kegeles SM. The Development of a Counselling-Based HIV Prevention Intervention for African American Men Who Have Sex With Men and Women: The Bruthas Project. *AIDS Educ Prev* 2015;27(6):505-21.
2. Arulogun OS, Hurst S, Owolabi MO, Akinyemi RO, Uvere E, Saulson R, Ovbiagele B. Experience of Using an Interdisciplinary Task Force to Develop a Culturally Sensitive Multipronged Tool to Improve Stroke Outcomes in Nigeria. *eNeurologicalSci* 2016;4:10-14.
3. Asher L, Fekadu A, Hanlon C, Mideksa G, Eaton J, Patel V, De Silva MJ. Development of a Community-Based Rehabilitation Intervention for People with Schizophrenia in Ethiopia. *PLoS One* 2015;10(11): e0143572.
4.* Avis JL, Cave AL, Donaldson S, Ellendt C, Holt NL, Jelinski S, Martz P, Maximova K, Padwal R, Wild TC, Ball GD. Working With Parents to Prevent Childhood Obesity: Protocol for a Primary Care-Based eHealth Study. *JMIR Res Protoc* 2015;4(1):e35.
5. Avis JL, Holt NL, Maximova K, van Mierlo T, Fournier R, Padwal R, Cave AL, Martz P, Ball GD. The Development and Refinement of an e-Health Screening, Brief Intervention, and Referral to Treatment for Parents to Prevent Childhood Obesity in Primary Care. *Telemed J E Health* 2016;22(5):385-94.
6. Bailey JV, Webster R, Hunter R, Griffin M, Freemantle N, Rait G, Estcourt C, Michie S, Anderson J, Stephenson J, Gerressu M, Ang CS, Murray E. The Men's Safer Sex project: intervention development and feasibility randomised controlled trial of an interactive digital intervention to increase condom use in men. *Health Technol Assess* 2016;20(91):1-124.
7. Bazzi AR, Fergus KB, Stephenson R, Finneran CA, Coffey-Esquivel J, Hidalgo MA, Hoehnle S, Sullivan PS, Garofalo R, Mimiaga MJ. A Dyadic Behavioral Intervention to Optimize Same Sex Male Couples' Engagement Across the HIV Care Continuum: Development of and Protocol for an Innovative Couples-based Approach (Partner Steps). JMIR Res Protoc 2016;5(3):e168.
8. Brach JS, Francois SJ, VanSwearingen JM, Gilmore S, Perera S, Studenski SA. Translation of a Motor Learning Walking Rehabilitation Program Into a Group-Based Exercise Program for Community-Dwelling Older Adults. *PM R* 2016;8(6):520-8.
9. Bryant K, Moore T, Willis N, Hadden K. Development of a Faith-Based Stress Management Intervention in a Rural African American Community. *Prog Community Health Partnersh* 2015;9(3):423-30.
10. Buis LR, Artinian NT, Schwiebert L, Yarandi H, Levy PD. Text Messaging to Improve Hypertension Medication Adherence in African Americans: BPMED Intervention Development and Study Protocol. *JMIR Res Protoc* 2015;4(1):e1.
11.* Cadogan CA, Ryan C, Francis JJ, Gormley GJ, Passmore P, Kerse N, Hughes CM. Development of an intervention to improve appropriate polypharmacy in older people in primary care using a theory-based method. *BMC Health Serv Res* 2016;16(1):661.

12. Chacko A, Isham A, Cleek AF, McKay MM. Using mobile health technology to improve behavioral skill implementation through homework in evidence-based parenting intervention for disruptive behavior disorders in youth: study protocol for intervention development and evaluation. *Pilot Feasibility Stud* 2016;2:57.

13.* Charles P, Gorman-Smith D, Jones A. Designing an Intervention to Promote Child Development Among Fathers With Antisocial Behavior. *Res Soc Work Pract* 2016;26(1):20-27.

14. Choo E, Guthrie KM, Mello M, Wetle TF, Ranney M, Tapé C, Zlotnick C. "I need to hear from women who have 'been there'": Developing a woman-focused intervention for drug use and partner violence in the emergency department. *Partner Abuse* 2016; 7(2):193-220.

15. Clarkesmith DE, Pattison HM, Borg Xuereb C, Lane DA. Developing a Complex Educational-Behavioural Intervention: The TREAT Intervention for Patients with Atrial Fibrillation. *Healthcare (Basel)* 2016;4(1):E10.

16.* Connell LA, McMahon NE, Redfern J, Watkins CL, Eng JJ. Development of a behaviour change intervention to increase upper limb exercise in stroke rehabilitation. *Implement Sci* 2015;10:34.

17. Corder K, Schiff A, Kesten JM, van Sluijs EM. Development of a universal approach to increase physical activity among adolescents: the GoActive intervention. *BMJ Open* 2015;5(8):e008610.

18. Cross-Bardell L, George T, Bhoday M, Tuomainen H, Qureshi N, Kai J. Perspectives on enhancing physical activity and diet for health promotion among at-risk urban UK South Asian communities: a qualitative study. *BMJ Open* 2015;5(2):e007317.

19. Curtis KE, Lahiri S, Brown KE. Targeting Parents for Childhood Weight Management: Development of a Theory-Driven and User-Centered Healthy Eating App. *JMIR Mhealth Uhealth* 2015;3(2):e69.

20. Davies E, Martin J, Foxcroft D. Development of an adolescent alcohol misuse intervention based on the Prototype Willingness Model: A Delphi study. *Health Educ* 2016;116(3):275-291.

21. de Visser RO, Graber R, Hart A, Abraham C, Memon A, Watten P, Scanlon T. Using qualitative methods within a mixed-methods approach to developing and evaluating interventions to address harmful alcohol use among young people. *Health Psychol* 2015;34(4):349-60.

22. Enah C, Piper K, Moneyham L. Qualitative evaluation of the relevance and acceptability of a web-based HIV prevention game for rural adolescents. *J Pediatr Nurs* 2015;30(2):321-8.

23. Ewing G, Ngwenya N, Benson J, Gilligan D, Bailey S, Seymour J, Farquhar M. Sharing news of a lung cancer diagnosis with adult family members and friends: a qualitative study to inform a supportive intervention. *Patient Educ Couns* 2016;99(3):378-385.

24.* Ford JA, Jones AP, Wong G, Clark AB, Porter T, Shakespeare T, Swart AM, Steel N. Improving access to high-quality primary care for socioeconomically disadvantaged older people in rural areas: a mixed method study protocol. *BMJ Open* 2015;5(9):e009104.

25. Foster C, Calman L, Grimmett C, Breckons M, Cotterell P, Yardley L, Joseph J, Hughes S, Jones R, Leonidou C, Armes J, Batehup L, Corner J, Fenlon D, Lennan E, Morris C, Neylon A, Ream E, Turner L, Richardson A. Managing fatigue after cancer treatment: development of RESTORE, a web-based resource to support self-management. *Psychooncology* 2015;24(8):940-9.

26. Free C, McCarthy O, French RS, Wellings K, Michie S, Roberts I, Devries K, Rathod S, Bailey J, Syred J, Edwards P, Hart G, Palmer M, Baraitser P. Can text messages increase safer sex behaviours in young people? Intervention development and pilot randomised controlled trial. *Health Technol Assess* 2016;20(57):1-82.

27. Geraghty AW, Muñoz RF, Yardley L, Mc Sharry J, Little P, Moore M. Developing an Unguided Internet-Delivered Intervention for Emotional Distress in Primary Care Patients: Applying Common Factor and Person-Based Approaches. *JMIR Ment Health* 2016;3(4):e53.

28. Giebel CM, Challis D, Hooper NM, Ferris S. A step-by-step translation of evidence into a psychosocial intervention for everyday activities in dementia: a focus group study. *Aging Ment Health* 2018;22(3):323-329.

29. Githinji S, Jones C, Malinga J, Snow RW, Talisuna A, Zurovac D. Development of a text-messaging intervention to improve treatment adherence and post-treatment review of children with uncomplicated malaria in western Kenya. *Malar J* 2015;14:320.

30. Goldberg J, Hinchey J, Feder S, Schulman-Green D. Developing and Evaluating a Self-Management Intervention for Women With Breast Cancer. *West J Nurs Res* 2016;38(10):1243-63.

31.* Golin CE, Knight K, Carda-Auten J, Gould M, Groves J, L White B, Bradley-Bull S, Amola K, Fray N, Rosen DL, Mugavaro MJ, Pence BW, Flynn PM, Wohl D. Individuals motivated to participate in adherence, care and treatment (imPACT): development of a multi-component intervention to help HIV-infected recently incarcerated individuals link and adhere to HIV care. *BMC Public Health* 2016;16:935.

32.* Gray-Burrows KA, Day PF, Marshman Z, Aliakbari E, Prady SL, McEachan RR. Using intervention mapping to develop a home-based parental-supervised toothbrushing intervention for young children. *Implement Sci* 2016;11:61.

33.* Handley MA, Harleman E, Gonzalez-Mendez E, Stotland NE, Althavale P, Fisher L, Martinez D, Ko J, Sausjord I, Rios C. Applying the COM-B model to creation of an IT-enabled health coaching and resource linkage program for low-income Latina moms with recent gestational diabetes: the STAR MAMA program. *Implement Sci* 2016;11(1):73.

34. Hartlieb KB, Naar S, Ledgerwood DM, Templin TN, Ellis DA, Donohue B, Cunningham PB. Contingency management adapted for African-American adolescents with obesity enhances youth weight loss with caregiver participation: a multiple baseline pilot study. *Int J Adolesc Med Health* 2015;29(3):pii.

35.* Heath G, Cooke R, Cameron E. A Theory-Based Approach for Developing Interventions to Change Patient Behaviours: A Medication Adherence Example from Paediatric Secondary Care. *Healthcare (Basel)* 2015;3(4):1228-42.

36. Hindin MJ, Rodriguez MI, Gonsalves L, Say L. Adolescent health experience after abortion or delivery (AHEAD) trial: formative protocol for intervention development to prevent rapid, repeat pregnancy. *Reprod Health* 2015;12:111.

37.* Ingholt L, Sørensen BB, Andersen S, Zinckernagel L, Friis-Holmberg T, Frank VA, Stock C, Tjørnhøj-Thomsen T, Rod MH. How can we strengthen students' social relations in order to reduce school dropout? An intervention development study within four Danish vocational schools. BMC Public Health 2015;15:502.

38.* Katz ML, Paskett ED. The process of engaging members from two underserved populations in the development of interventions to promote the uptake of the HPV vaccine. *Health Promot Pract* 2015;16(3):443-53.

39. Krukowski RA, Hare ME, Talcott GW, Johnson KC, Richey PA, Kocak M, Balderas J, Colvin L, Keller PL, Waters TM, Klesges RC. Dissemination of the Look AHEAD intensive lifestyle intervention in the United States Air Force: study rationale, design and methods. *Contemp Clin Trials* 2015;40:232-9.

40. Lachman JM, Sherr LT, Cluver L, Ward CL, Hutchings J, Gardner F. Integrating Evidence and Context to Develop a Parenting Program for Low-Income Families in South Africa. *J Child Fam Stud* 2016;25(7):2337-2352.

41. Laine H, Araújo-Soares V, Haukkala A, Hankonen N. Acceptability of Strategies to Reduce Student Sitting: A Mixed-Methods Study With College Teachers. *Health Promot Pract* 2017;18(1):44-53.

42.* Mackenzie K, Goyder E, Eves F. Acceptability and feasibility of a low-cost, theory-based and co-produced intervention to reduce workplace sitting time in desk-based university employees. *BMC Public Health* 2015;15:1294.

43.* Marsac ML, Winston FK, Hildenbrand AK, Kohser KL, March S, Kenardy J, Kassam-Adams N. Systematic, theoretically-grounded development and feasibility testing of an innovative, preventive web-based game for children exposed to acute trauma. *Clin Pract Pediatr Psychol* 2015;3(1):12-24.

44.* Martin MA, Floyd EC, Nixon SK, Villalpando S, Shalowitz M, Lynch E. Asthma in Children With Comorbid Obesity: Intervention Development in a High-Risk Urban Community. *Health Promot Pract* 2016;17(6):880-890.

45.* McMillen JC, Narendorf SC, Robinson D, Havlicek J, Fedoravicius N, Bertram J, McNelly D. Development and piloting of a treatment foster care program for older youth with psychiatric problems. Child Adolesc Psychiatry Ment Health 2015;9:23.

46. Merlin JS, Young SR, Johnson MO, Saag M, Demonte W, Modi R, Shurbaji S, Anderson WA, Kerns R, Bair MJ, Kertesz S, Davies S, Turan JM. Using Patient Perspectives to Inform the Development of a Behavioral Intervention for Chronic Pain in Patients with HIV: A Qualitative Study. *Pain Med* 2017;18(5):879-888.

47. Miller SM, Hudson SV, Hui SK, Diefenbach MA, Fleisher L, Raivitch S, Belton T, Roy G, Njoku A, Scarpato J, Viterbo R, Buyyounouski M, Denlinger C, Miyamoto C, Reese A, Baman J. Development and preliminary testing of PROGRESS: a Web-based education program for prostate cancer survivors transitioning from active treatment. *J Cancer Surviv* 2015;9(3):541-53.

48. Moreau M, Gagnon MP, Boudreau F. Development of a fully automated, web-based, tailored intervention promoting regular physical activity among insufficiently active adults with type 2 diabetes: integrating the I-change model, self-determination theory, and motivational interviewing components. *JMIR Res Protoc* 2015;4(1):e25.

49.* Morrison D, Mair FS, Chaudhuri R, McGee-Lennon M, Thomas M, Thomson NC, Yardley L, Wyke S. Details of development of the resource for adults with asthma in the RAISIN (randomized trial of an asthma internet self-management intervention) study. *BCM Med Inform Decis Mak* 2015;15:57.

50.* Mummah SA, King AC, Gardner CD, Sutton S. Iterative development of Vegethon: a theory-based mobile app intervention to increase vegetable consumption. *Int J Behav Nutr Phys Act* 2016;13:90.

51. Muramoto ML, Matthews E, Ritenbaugh CK, Nichter MA. Intervention development for integration of conventional tobacco cessation interventions into routine CAM practice. *BMC Complement Altern Med* 2015;15:96.

52. Murchie P, Allan JL, Brant W, Dennis M, Hall S, Masthoff J, Walter FM, Johnston M. Total skin self-examination at home for people treated for cutaneous melanoma: development and pilot of a digital intervention. *BMJ Open* 2015;5(8): e007993.

53.* Njeru JW, Patten CA, Hanza MM, Brockman TA, Ridgeway JL, Weis JA, Clark MM, Goodson M, Osman A, Porraz-Capetillo G, Hared A, Myers A, Sia IG, Wieland ML. Stories for change: development of a diabetes digital storytelling intervention for refugees and immigrants to minnesota using qualitative methods. *BMC Public Health* 2015;15:1311.

54. Noble AJ, Marson AG, Tudur-Smith C, Morgan M, Hughes DA, Goodacre S, Ridsdale L. 'Seizure First Aid Training' for people with epilepsy who attend emergency departments, and their family and friends: study protocol for intervention development and a pilot randomised controlled trial. *BMJ Open* 2015;5(7): e009040.

55.* O'Connell SE, Jackson BR, Edwardson CL, Yates T, Biddle SJ, Davies MJ, Dunstan D, Esliger D, Gray L, Miller P, Munir F. Providing NHS staff with height-adjustable workstations and behaviour change strategies to reduce workplace sitting time: protocol for the Stand More AT (SMArT) Work cluster randomised controlled trial. *BMC Public Health* 2015;15:1219.

56. Owens C, Charles N. Implementation of a text-messaging intervention for adolescents who self-harm (TeenTEXT): a feasibility study using normalisation process theory. *Child Adolesc Psychiatry Ment Health* 2016;10:14.
57. Panda S, Das RS, Maruf SKA, Pahari S. Exploring Stigma in Low HIV Prevalence Settings in Rural West Bengal, India: Identification of Intervention Considerations. *J Mix Methods Res* 2015;9(4):362-385.
58. Phelps C, Minou M, Baker A, Hughes C, French H, Hawkins W, Leeuwenberg A, Crabtree R, Hutchings PB. Necessary but not sufficient? Engaging young people in the development of an avatar-based online intervention designed to provide psychosocial support to young people affected by their own or a family member's cancer diagnosis. *Health Expect* 2017;20(3):459-470.
59.* Poleshuck E, Mazzotta C, Resch K, Rogachefsky A, Bellenger K, Raimondi C, Thompson Stone J, Cerulli C. Development of an Innovative Treatment Paradigm for Intimate Partner Violence Victims With Depression and Pain Using Community-Based Participatory Research. *J Interpers Violence* 2016;33(17):2704-2724.
60. Quinn L, Trubey R, Gobat N, Dawes H, Edwards RT, Jones C, Townson J, Drew C, Kelson M, Poile V, Rosser A, Hood K, Busse M. Development and Delivery of a Physical Activity Intervention for People With Huntington Disease: Facilitating Translation to Clinical Practice. *J Neurol Phys Ther* 2016;40(2):71-80.
61. Ramaswamy M, Simmons R, Kelly PJ. The development of a brief jail-based cervical health promotion intervention. *Health Promot Pract* 2015;16(3):432-42.
62. Ramirez M, Toussaint M, Woods-Jaeger B, Harland K, Wetjen K, Wilgenbusch T, Pitcher G, Jennissen C. Link for Injured Kids: A Patient-Centered Program of Psychological First Aid After Trauma. *Pediatr Emerg Care* 2015;00(00).
63. Reisner SL, Hughto JM, Pardee DJ, Kuhns L, Garofalo R, Mimiaga MJ. LifeSkills for Men (LS4M): Pilot Evaluation of a Gender-Affirmative HIV and STI Prevention Intervention for Young Adult Transgender Men Who Have Sex with Men. *J Urban Health* 2016;93(1):189-205.
64.* Rothman EF, Wang N. A feasibility test of a brief motivational interview intervention to reduce dating abuse perpetration in a hospital setting. *Psychol Violence* 2016;6(3):433-441.
65. Sadler E, Sarre S, Tinker A, Bhalla A, McKevitt C. Developing a novel peer support intervention to promote resilience after stroke. *Health Soc Care Community* 2017;25(5):1590-1600.
66. Salazar AM, Haggerty KP, Roe SS. Fostering Higher Education: A Postsecondary Access and Retention Intervention for Youth with Foster Care Experience. *Child Youth Serv Rev* 2016;70:46-56.
67. Sibley KM1, Brooks D, Gardner P, Janaudis-Ferreira T, McGlynn M, O'Hoski S, McEwen S, Salbach NM, Shaffer J, Shing P, Straus SE, Jaglal SB. Development of a Theory-Based Intervention

to Increase Clinical Measurement of Reactive Balance in Adults at Risk of Falls. *J Neurol Phys Ther* 2016;40(2):100-6.

68.* Simonsen SE, Digre KB, Ralls B, Mukundente V, Davis FA, Rickard S, Tavake-Pasi F, Napia EE, Aiono H, Chirpich M, Stark LA, Sunada G, Keen K, Johnston L, Frost CJ, Varner MW, Alder SC. A gender-based approach to developing a healthy lifestyle and healthy weight intervention for diverse Utah women. *Eval Program Plann* 2015;51:8-16.

69.* Sinnott C, Mercer SW, Payne RA, Duerden M, Bradley CP, Byrne M. Improving medication management in multimorbidity: development of the MultimorbiditY COllaborative Medication Review And DEcision Making (MY COMRADE) intervention using the Behaviour Change Wheel. *Implement Sci* 2015;10:132.

70. Smallwood SW, Freedman DA, Pitner RO, Sharpe PA, Cole JA, Hastie S, Hunter B. Implementing a Community Empowerment Center to Build Capacity for Developing, Implementing, and Sustaining Interventions to Promote Community Health. *J Community Health* 2015;40(6):1122-9.

71. Smith SA, Whitehead MS, Sheats JQ, Fontenot B, Alema-Mensah E, Ansa B. Formative research to develop a lifestyle application (app) for African American breast cancer survivors. *J Ga Public Health Assoc* 2016;6(1):50-59.

72.* Steinmo SH, Michie S, Fuller C, Stanley S, Stapleton C, Stone SP. Bridging the gap between pragmatic intervention design and theory: using behavioural science tools to modify an existing quality improvement programme to implement "Sepsis Six". *Implement Sci* 2016;11:14.

73.* Sturgiss EA, Douglas K. A collaborative process for developing a weight management toolkit for general practitioners in Australia-an intervention development study using the Knowledge To Action framework. *Pilot Feasibility Stud* 2016;2:20.

74. Sun V, Kim JY, Raz DJ, Chang W, Erhunmwunsee L, Uranga C, Ireland AM, Reckamp K, Tiep B, Hayter J, Lew M, Ferrell B, McCorkle R. Preparing Cancer Patients and Family Caregivers for Lung Surgery: Development of a Multimedia Self-Management Intervention. *J Cancer Educ* 2016;33(3):557-563.

75. Sun V, Kim J, Kim JY, Raz DJ, Merchant S, Chao J, Chung V, Jimenez T, Wittenberg E, Grant M, Ferrell B. Dietary alterations and restrictions following surgery for upper gastrointestinal cancers: Key components of a health-related quality of life intervention. *Eur J Oncol Nurs* 2015;19(4):343-8.

76. Tarzia L, Murray E, Humphreys C, Glass N, Taft A, Valpied J, Hegarty K. I-DECIDE: An Online Intervention Drawing on the Psychosocial Readiness Model for Women Experiencing Domestic Violence. *Womens Health Issues* 2016;26(2):208-16.

77. Tavender EJ, Bosch M, Gruen RL, Green SE, Michie S, Brennan SE, Francis JJ, Ponsford JL, Knott JC, Meares S, Smyth T, O'Connor DA. Developing a targeted, theory-informed implementation intervention using two theoretical frameworks to address health professional and organisational

factors: a case study to improve the management of mild traumatic brain injury in the emergency department. *Implement Sci* 2015;10:74.

78.* Theeke LA, Mallow JA. The Development of LISTEN: A Novel Intervention for Loneliness. *Open J Nurs* 2015;5(2):136-143.

79. Tomkins-Lane CC, Lafave LM, Parnell JA, Rempel J, Moriartey S, Andreas Y, Wilson PM, Hepler C, Ray HA, Hu R. The spinal stenosis pedometer and nutrition lifestyle intervention (SSPANLI): development and pilot. *Spine J* 2015;15(4):577-86.

80. van Hoek E, Bouwman LI, Koelen MA, Lutt MAJ, Feskens EJM, Janse AJ. Development of a Dutch intervention for obese young children. *Health Promot Int* 2017;32(4):624-635.

81.* Vaughn LM, Jacquez F, Marschner D, McLinden D. See what we say: using concept mapping to visualize Latino immigrant's strategies for health interventions. *Int J Public Health* 2016;61(7):837-45.

82. Villadsen SF, Mortensen LH, Andersen AM. Care during pregnancy and childbirth for migrant women: How do we advance? Development of intervention studies--the case of the MAMAACT intervention in Denmark. *Best Pract Res Clin Obstet Gynaecol* 2016;32:100-12.

83. Webb J, Foster J, Poulter E. Increasing the frequency of physical activity very brief advice for cancer patients. Development of an intervention using the behaviour change wheel. *Public Health* 2016;133:45-56.

84. Webster R, Michie S, Estcourt C, Gerressu M, Bailey JV, MenSS Trial Group. Increasing condom use in heterosexual men: development of a theory-based interactive digital intervention. *Transl Behav Med* 2016;6(3):418-27.

85.* Withall J, Haase AM, Walsh NE, Young A, Cramp F. Physical activity engagement in early rheumatoid arthritis: a qualitative study to inform intervention development. *Physiotherapy* 2016;102(3):264-71.

86.* Xie A, Carayon P, Cox ED, Cartmill R, Li Y, Wetterneck TB, Kelly MM. Application of participatory ergonomics to the redesign of the family-centred rounds process. *Ergonomics* 2015;58(10):1726-44.

87. Ybarra ML, Prescott TL, Philips GL, Bull SS, Parsons JT, Mustanski B. Iteratively Developing an mHealth HIV Prevention Program for Sexual Minority Adolescent Men. AIDS Behav 2016;20(6):1157-72.
